# Studies of New Iridium Catalysts Supported on Modified Silicalite-1—Their Structure and Hydrogenating Properties

**DOI:** 10.3390/ma14164465

**Published:** 2021-08-09

**Authors:** Michał Zieliński, Monika Kot, Mariusz Pietrowski, Robert Wojcieszak, Jolanta Kowalska-Kuś, Ewa Janiszewska

**Affiliations:** 1 Faculty of Chemistry, Adam Mickiewicz University in Poznań, Uniwersytetu Poznańskiego 8, 61-614 Poznań, Poland; monika.kot@amu.edu.pl (M.K.); mariop@amu.edu.pl (M.P.); jolakow@amu.edu.pl (J.K.-K.); 2 Université Lille, CNRS, Centrale Lille, Université Artois, UMR 8181-UCCS-Unité de Catalyse et Chimie du Solide, F-59000 Lille, France; robert.wojcieszak@univ-lille.fr

**Keywords:** iridium catalyst, modified silicalite-1, surface acidity, toluene hydrogenation

## Abstract

This paper investigates the catalytic properties of the iridium catalysts supported on modified silicalite-1. Post-synthesis modification of silicalite-1, with solutions of ammonium compounds (NH_4_F and NH_4_OH), appeared to be an efficient method to generate the acidic sites in starting support. The modification of support led not only to changes in its acidity but also its porosity—formation of additional micro- and mesopores. The novel materials were used as supports for iridium. The iridium catalysts (1 wt.% Ir) were characterized by N_2_ adsorption/desorption measurements, temperature-programmed reduction with hydrogen (TPR-H_2_), H_2_ chemisorption, transmission electron microscopy (TEM), temperature-programmed desorption of ammonia (TPD-NH_3_), X-ray photoelectron spectroscopy (XPS) and tested in the hydrogenation of toluene reaction. The catalytic activity of iridium supported on silicalite-1 treated with NH_4_OH (higher porosity of support, better dispersion of active phase) was much higher than that of Ir supported on unmodified and modified with NH_4_F silicalite-1.

## 1. Introduction

Dearomatization has become an important subject in environmental catalysis worldwide to obtain clean fuels. The presence of aromatics in fuel reduces the cetane number in diesel and enhances the emission of particulate matter which is known to be responsible for various health problems when inhaled. Therefore, environmental regulations are directed to decrease these emissions from vehicle exhausts. Moreover, the aromatic reduction of 10 wt.% results in cetane number increase by ~3 allowing better engine operation [1]. The removal of aromatic compounds from gasoline or diesel is realized by catalytic hydrogenation [2].

Noble metal catalysts, which work at relatively low temperatures, are important for clean fuel production by aromatic saturation. However, these catalysts are very sensitive to the sulfur- and nitrogen-containing compounds presented in the feedstock [3]. The hydrodearomatization activity and sulfur-tolerance of noble-metals catalysts may be enhanced by deposition of noble metal (Pt, Pd, etc.) on acidic supports such as zeolite H-ZSM-5, ultrastable zeolite Y (USY), H-beta, mordenite, or alumina [4,5,6,7]. These effects are caused by the synergistic effects between noble metal particles and acid sites of support. The electron transfers partially from the metal particles to the acid sites that lead to the formation of electron-deficient metal (Me^δ+^), which decreases the strength of the sulfur-metal bond preventing the formation of metal sulfides and the noble metal particles (active sites) become more stable [8]. Moreover, the acid sites of the support provide additional active sites on which aromatic and sulfur compounds are hydrogenated by the spillover hydrogen from the metal surface [9]. It was shown that noble metals supported on zeolites are more active than supported on alumina. On the other hand, a high acidity of zeolites also favors excessive cracking and coke formation causing quick deactivation of the catalysts [10,11]. No deactivation was observed when the metals were supported on alumina or SiO_2_–TiO_2_, the supports with weak and intermediate acid sites [12,13].

It was shown that treatment of siliceous zeolites with a MFI structure as well as amorphous silica with an aqueous solution of ammonium salts and at least one basic compound generates their additional acidity [14,15,16,17]. The Fourier-transform infrared spectroscopy (FT-IR) indicated the creation of H-bonded silanol defect groups of different kinds with some acidity in the samples modified in this way. It caused also the generation of mesopores by partial leaching of silicon from the framework during the modification procedure [16]. It allows decreasing the diffusion limitation if they are used as catalysts or support of catalysts.

Within the above background, the purpose of this work was to use the silicalite materials with MFI structure (silicalite-1) with different acidity as supports of iridium catalysts. The acidic sites in pristine silicalite-1 were generated employing post-synthesis treatment with solutions of various ammonium agents (NH_4_F, NH_4_OH) at elevated temperature [16]. The influence of support acidity on the efficiency of the resulting iridium catalysts for toluene hydrogenation was examined. Toluene was used as a model compound to simulate the aromatics in diesel fuels because the hydrogenation of toluene is known to be more difficult than that of benzene and naphthalene [18]. Taking into account the influence of texture and the acid function of supports on the activity of such catalysts, the characterization of the supports and catalysts was performed using Brunauer–Emmet–Teller (BET) method, temperature-programmed reduction with hydrogen (TPR-H_2_), H_2_ chemisorption, transmission electron microscopy (TEM), temperature-programmed desorption of ammonia (TPD-NH_3_), and X-ray photoelectron spectroscopy (XPS). The catalytic activity of iridium catalysts supported on unmodified and modified silicalite-1 was compared. The impact of texture and acidity of catalysts on the activity of iridium catalysts was assessed in the reaction of toluene hydrogenation. To the best of our knowledge, no data are available on such modifications of silicalite-1 followed by the application of resulting material as iridium catalyst support for hydrogenation processes.

## 2. Materials and Methods

Silicalite-1 (denoted as Sil) was prepared according to our earlier procedure [16]. The calcined silicalite-1 was modified using 1 M solutions of various ammonium compounds (NH_4_OH or NH_4_F, Aldrich, Saint Louis, MO, USA). The sample (1 g) of silicalite-1 was mixed with the respective NH_4_^+^ source solution (100 cm^3^). The mixture (the silicalite-1 and aqueous solution of ammonium compounds) was stirred under reflux at 60 °C for 1 h. After the treatment, the samples were filtered, washed with deionized water, dried at 105 °C for 24 h, and then calcined in air (Linde, Pullach im Isartal, Germany) at 550 °C for 3 h with a temperature ramp of 5 °C⋅min^−1^. The resulting samples were labelled as Sil-X, where X stands for the anion (OH^−^ or F^−^) of applied ammonium compounds (e.g., SilOH was prepared with 1 M NH_4_OH solution). Removal of fluorine and decomposition of ammonium cations in modified silicalite-1 was confirmed by means of energy dispersive X-ray spectroscopy (EDS analysis) (Thermo Fisher Scientific, Waltham, MA, USA, with an Ultra Dry Silicon Drift X-ray Detector), X-ray photoelectron spectra (XPS, Ultra High Vacuum (UHV) System (Specs, Berlin, Germany)), and X-ray fluorescence (XRF, Mini-Pal 2 spectrometer (Panalytical, Malvern, Worcestershire, UK)).

The Ir/support (support = Sil, Sil-OH or Sil-F; content of Ir was 1 wt.%) catalysts were prepared by the conventional impregnation method using hexachloroiridium (IV) acid hydrate (H_2_IrCl_6_⋅× H_2_O, 99.995%, Aldrich, Saint Louis, MO, USA) as a metal precursor. An appropriate amount of support was placed in an aqueous solution of H_2_IrCl_6_⋅× H_2_O followed by evaporation on a rotary evaporator (Heidolph, Schwabach/Nuremberg, Germany). Then the supports loaded with precursors were dried at 105 °C for 24 h and labelled as Ir/Sil-D, Ir/Sil-OH-D, and Ir/Sil-F-D.

Before measurements of hydrogen chemisorption, low-temperature nitrogen adsorption-desorption (BET surface area and porosity), determination of metal content (inductively coupled plasma optical emission spectrometry, ICP-OES), temperature-programmed desorption of ammonia (TPD-NH_3_), X-ray photoelectron spectroscopy (XPS), transmission electron microscopy (TEM), and catalytic activity, precursor-impregnated supports (Ir/Sil-D, Ir/Sil-OH-D, and Ir/Sil-F-D) were reduced in hydrogen flow (99.99%, Linde, Pullach im Isartal, Germany, 50 cm^3^⋅min^−1^) with a temperature ramp of 10 °C⋅min^−1^. After reaching the setpoint (400 °C), the catalysts reduction was continued for 2 h. The reduced catalysts were labelled as Ir/Sil, Ir/Sil-OH, and Ir/Sil-F.

The Brunauer–Emmet–Teller (BET) surface areas were determined by N_2_ adsorption-desorption at −196 °C using a Micromeritics ASAP 2010 sorptometer (Micromeritics, Norcross, GA, USA). Total pore volume and average pore diameter were determined by BET method and by applying the Barrett–Joyner–Halenda (BJH) method to the desorption branch of the isotherm. The microporous and mesoporous surface area were determined from t-plot method. Prior to the measurements, the samples were outgassed at 225 °C.

Powder X-ray diffraction patterns of supports and catalysts were recorded on the Philips Bruker D8 Advance diffractometer (Billerica, MA, USA) using Cu Kα radiation (λ = 1.54056 Å).

The metal loading in the catalysts after reduction (H_2_; 400 °C; 2 h) was determined by ICP-OES method (Inductively Coupled Plasma Optical Emission Spectroscopy) on a Varian Vista-MPX spectrometer (Palo Alto, CA, USA).

Measurements of temperature-programmed reduction with hydrogen (TPR-H_2_) were carried out on Pulse ChemiSorb 2705 (Micromeritics, Norcross, GA, USA) instrument. The hydrogen consumption was monitored with a thermal conductivity detector (TCD) and the signal was normalized to the same sample weight—100 mg. Detailed experimental procedure of TPR-H_2_ analysis is presented in the Supplementary Materials.

The temperature-programmed desorption of ammonia (TPD-NH_3_) measurements were carried out on Pulse ChemiSorb 2705 instrument (Micromeritics, Norcross, GA, USA) for the catalysts reduced at 400 °C for 2 h. The TPD-NH_3_ measurements of acidity were performed in a flow reactor. Detailed experimental procedure of TPD-NH_3_ analysis is presented in the Supplementary Materials.

Transmission electron microscope (TEM) images were recorded on a JEOL 2000 microscope (Akishima, Tokyo, Japan) operating at accelerating voltage of 80 kV. The average particle was calculated from 100 particles using ImageJ program (v.1.53e, 2020, National Institutes of Health and the Laboratory for Optical and Computational Instrumentation (LOCI, University of Wisconsin, Madison, WI, USA)).

The hydrogen chemisorption measurements were conducted by the static method at 35 °C on an ASAP 2010C sorptometer (Micromeritics, Norcross, GA, USA). Detailed experimental procedure of H_2_ chemisorption analysis is presented in [19] and in the Supplementary Materials.

X-ray photoelectron spectroscopy (XPS) analysis of supports and catalysts were carried out with an ultra-high vacuum (UHV) system (Specs, Berlin, Germany). The examined materials were irradiated with a monochromatized aluminum X-ray source (Al Kα (1486.6 eV)). The charge referencing method used was the C (C, H) component of the C 1s peak of adventitious carbon fixed at 284.6 eV. Spectroscopic data were processed by the CasaXPS ver. 2.3.17PR1.1 software (2016, Casa Software Ltd., Teignmouth, UK), using a peak-fitting routine with Shirley background.

Toluene hydrogenation was performed at atmospheric pressure using a fixed-bed flow reactor and H_2_ as carrier gas. Before the reaction was started, fresh catalyst (25 mg) was placed into the reactor and reduced in situ in a flow (100 cm^3^⋅min^−1^) of pure hydrogen (99.99%) at 400 °C for 2 h. The reaction mixture was prepared by passing hydrogen (50 cm^3^⋅min^−1^) through a saturator filled with toluene (Aldrich, Saint Louis, MO, USA) and the obtained gaseous mixture (toluene and hydrogen) was directed to the reactor. The concentration of toluene in the feed was stable and established to 0.75 μmol⋅cm^−3^. The catalytic activities were measured at temperatures range of 75–225 °C. Details of the hydrogenation reaction were presented in [17]. The scheme (Figure S1) of the setup used for toluene hydrogenation and detailed experimental procedure are also presented in Supplementary Materials. For selected samples, a stability test at 125 °C for 22 h was also performed. The products were analyzed every 10 min by a SRI gas chromatograph (SRI Instruments, Earl St. Torrance, CA, USA) equipped with a capillary column Restek MXT-1 and a TCD detector. The catalytic activity was expressed as an apparent rate calculated by the following equation [19]:

(1) $$r_{t}=\frac{FYC}{N}$$

(where *F*—total flow rate of feed (cm^3^⋅min^−1^); *Y*—fractional conversion; *C*—concentration of toluene in the feed (mol_Tl_⋅cm^−3^); and *N*—iridium content (mol_Ir_) in the sample) or as turnover frequency TOF (min^−1^) in moles of toluene reacted per surface Ir atoms (determined by hydrogen chemisorption).

## 3. Results

X-ray diffractograms of the supports and catalysts (Figure S2) confirmed the correct MFI structure of the support [16]. The crystallinity of the catalysts is comparable to the crystallinity of the supports. Characteristic peaks of metallic Ir species (2θ = 40.6° (111) and 47.3° (200)) were not observed due to the low iridium content (1 wt.%) as well as a high dispersion of iridium species on the supports [20].

**Figure 1:**
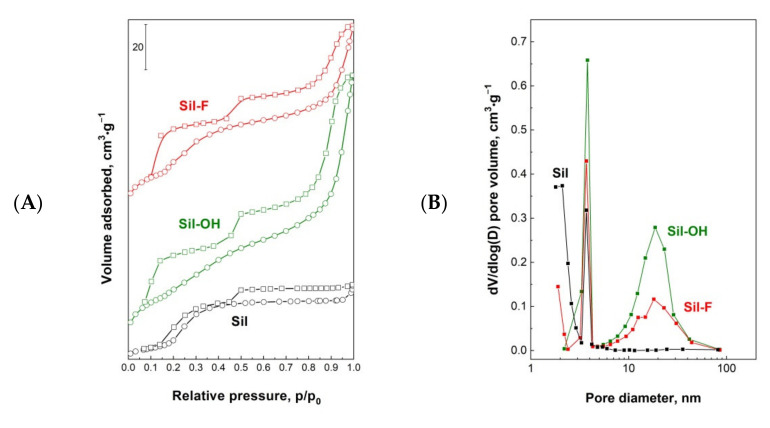

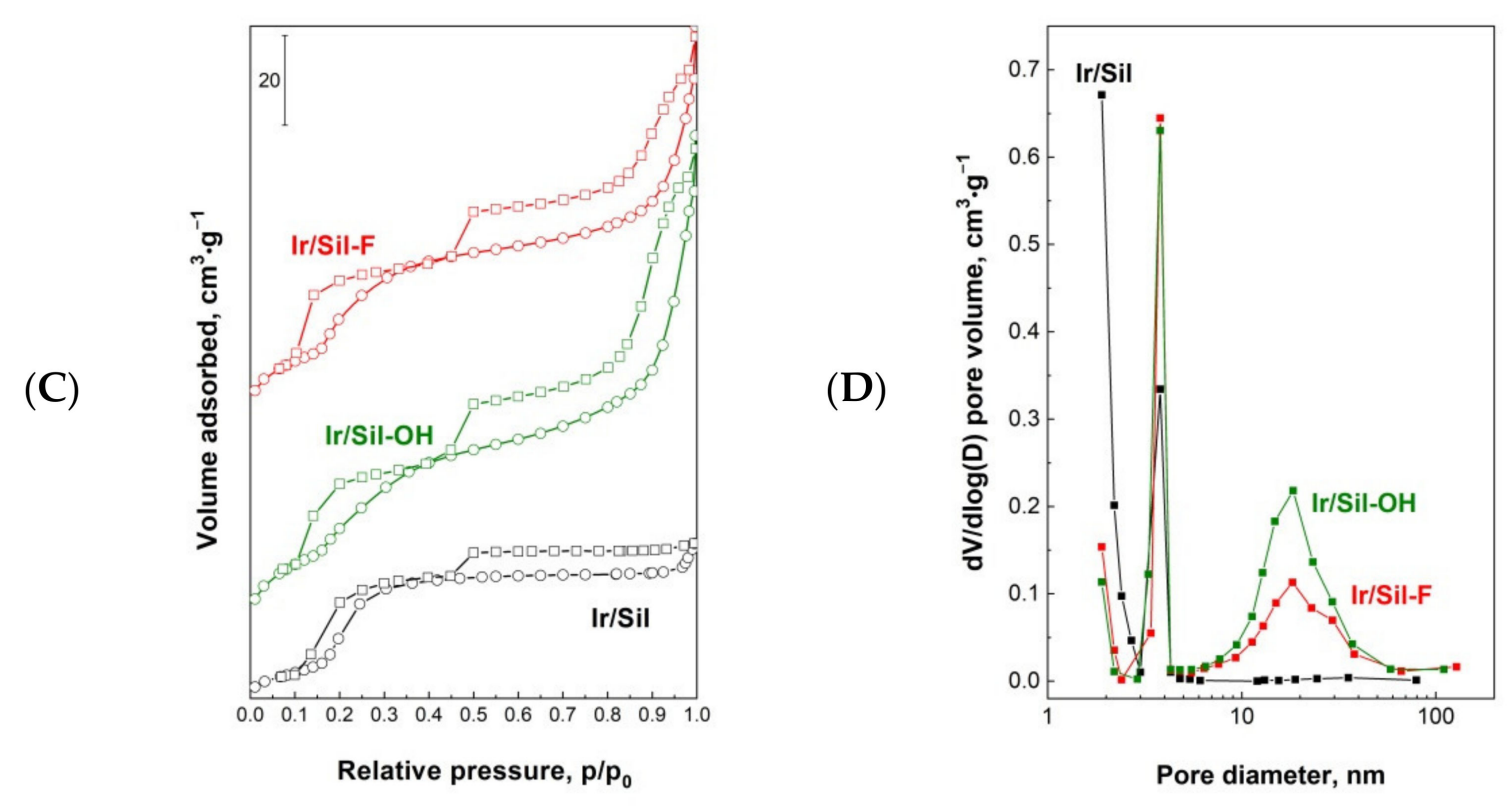
N_2_ adsorption-desorption isotherms and pore size distribution determined by Barrett–Joyner–Halenda method to the desorption branch of the isotherm for calcined supports (**A**,**B**) and reduced catalysts (**C**,**D**).

One of the important parameters characterizing micro- and mesoporous systems is their BET surface area and porosity. The textures of supports after calcination at 550 °C (Figure 1A,B, Table 1) and iridium catalysts after reduction at 400 °C (Figure 1C,D, Table 1) were characterized by the low-temperature nitrogen adsorption-desorption measurements.

**Figure 2:**
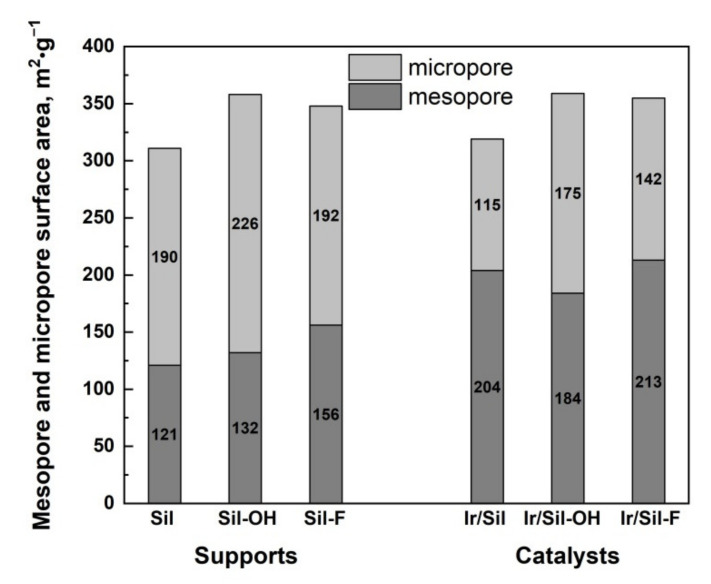
Micropore and mesopore surface area of calcined supports and iridium reduced catalysts.

The N_2_ adsorption-desorption curves of unmodified Sil are typical for Silicalite-1 materials presented in the literature [16,21] and correspond to type Ib (according to the International Union of Pure and Applied Chemistry (IUPAC) classification) characteristic for materials having pore size distributions over a broader range including wider micropores and possibly narrow mesopores (<2.5 nm) (Figure 1A,B, Table 1) [22]. The low-temperature N_2_ sorption isotherms exhibit two hysteresis loops. The first one at a low partial pressure p/p_0_ = 0.1–0.4 range is detected for highly silica and pure silica MFI materials and can be attributed to the defects in the structure [23]. The increasing volume of this hysteresis loop for modified samples can be explained by the formation of the additional framework defects as a result of the modification. The second hysteresis loop at p/p_0_ > 0.5 for the Sil is attributed to the inter-crystalline porosity. The modification of silicalite-1 with ammonium compounds solutions leads to an increase of the BET surface area for all supports regardless of the type of used ammonium agents. The increase of the total pore volume and average pore diameter was observed especially after the use of NH_4_OH—Table 1. This increase is due to the formation of an additional mesoporous structure. The pore volume of the Sil-OH sample is one and a half times higher in comparison to the pore volume of the unmodified sample. The growing second loop in isotherms of the modified samples can be explained by a generation of additional mesoporosity during the used procedure. As shown in Figure 1B, some mesoporosity, resulted from the inter-crystalline voids, is seen in the pristine silicalite-1 (Sil), whereas the modified samples show the presence of additional, well-defined pores with larger diameters compared to those of the starting material. The average pore sizes calculated by the BJH method are larger for modified supports and are 8.4 and 5.7 nm for Sil-OH and Sil-F samples, respectively—Figure 1. The low-temperature nitrogen adsorption-desorption isotherms were also used to calculate surface area, cumulative pore diameter, and cumulative pore volume of iridium catalysts—Table 1. During the preparation of catalysts, by the impregnation method, the active phase covers the surface of the support. Due to the low amount of introduced active phase, the impregnation with iridium precursor does not change the texture of the supports—Figure 1C,D. The BET surface area, pore volume, and average pore size are similar to these values obtained for the supports before impregnation (Table 1). The nitrogen adsorption isotherms (Figure 1C) and pore size distribution (Figure 1D) of the catalysts are almost similar to those of the supports. Only the surface of the micropores and mesopores changed. A decrease is observed in the microporous surface area with simultaneous increase of the mesoporous surface area in comparison to their values for supports. It may be related to the blocking of the smallest channels by the crystallites of the introduced active phase as well as the formation of additional mesoporosity by treatment with acidic iridium source—Figure 2. The decrease of the volume of low-partial pressure hysteresis loop for iridium catalysts can indicate that some parts of the defect sites participate in the deposition of the active phase on the surface of supports.

**Figure 3:**
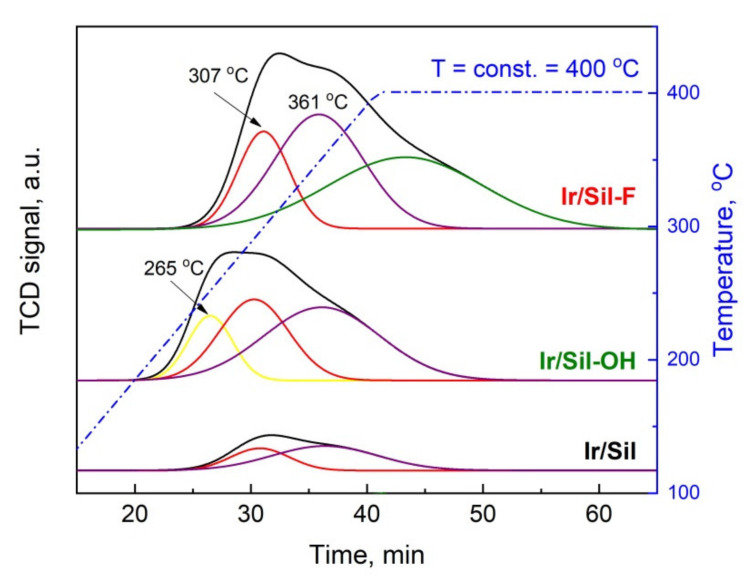
Temperature-programmed desorption of NH_3_ profiles with the Gaussian deconvolution of the reduced Ir/Sil samples. Signal intensity was normalized to 1 g of catalysts.

TPD-NH_3_ analysis allows determining not only the acid center concentration (Table 1) on the support or catalyst surface, but also estimating the distribution of their acid strength (Table 2). The TPD-NH_3_ profiles of the obtained catalysts and the results of their deconvolution were presented in Figure 3 and Table 2. The shape of the TPD-NH_3_ profiles indicates the presence of acid centers of different strengths—weak, medium, and strong. The desorption peaks at 265–310 °C range indicate the presence of weak centers, the peak with the maximum at about 360 °C corresponds to medium-strength centers, whereas from strong centers ammonia desorbed over 360 °C [24]. The maximum desorption temperature in our study was just 400 °C what was dictated by the temperature of the activation of iridium catalysts. Our previous studies (TPD-NH_3_ studies of supports [16]) showed a low acidity of the starting material. The TPD-NH_3_ profiles of the modified samples indicated the formation of additional acid sites as a result of the modification procedure. The number and the strength of created acid sites depended on the type of ammonium agents used for modification—Table 1.

The sample modified with NH_4_F indicated the highest overall acidity and the higher contribution of strong acid centers in comparison to the other samples. The sample modified with NH_4_OH shows lower total acidity in comparison to the acidity of Sil-F with a predominance of weak and moderate acid sites. The TPD-NH_3_ profiles of iridium catalysts allowed to calculate the concentration of surface acid centers of different strength (Table 2). The TPD-NH_3_ analysis indicated that the introduction of iridium phase on the surface of unmodified and modified silicalite-1 leads to a slight increase of the total acidity. The increase of total acidity of the supported noble metal catalysts in comparison to the acidity of the supports has been previously presented in the literature in the case of using metal chlorides or chlorometallic acids as a metal precursor. It was shown that the introduction of chlorides produced new acid sites [25].

The formation of defects playing the role of acid centers is also confirmed by the XPS measurements of the supports before and after the modification with ammonium compounds. In the Si 2p spectrum of the initial silicalite-1, only one signal was observed at binding energy BE = 103.55 eV. Modification with NH_4_OH as well as NH_4_F leads to an additional weak signal at a lower energy of ~101 eV. According to Alfonsetti et al. [26], this signal corresponds to SiO_x_, where x < 2. It indicates the partial removal of oxygen from the framework of silicalite-1 during modification and was confirmed by the calculated amount of oxygen and by the Si/O atomic ratio, which increases from the value of 0.66 for Sil by 0.67 for Sil-OH to 0.73 for Sil-F (Table 3). The formation of SiO_x_ species (x < 2) is also confirmed by the presence of an additional O 1s signal at BE~530.7 eV in the spectra of modified silicalites. The elimination of oxygen from the silicalite-1 framework results in the generation of oxygen vacancies, i.e., coordinated unsaturated silicon atoms that can act as Lewis acid centers.

**Figure 4:**
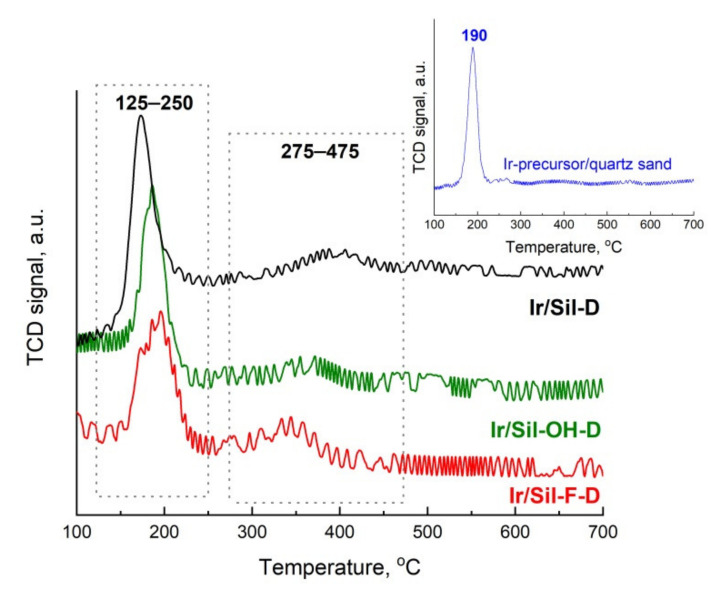
Temperature programmed reduction profiles of the dried catalyst precursors and iridium precursor supported on the quartz sand (inset). Signal intensity was normalized to 100 mg.

To obtain information on the reducibility of active phase precursors introduced into the supports studied, temperature-programmed reduction with hydrogen (TPR-H_2_) was performed. TPR-H_2_ measurements were carried out on fresh catalysts dried at 105 °C. No reduction signals were seen in profiles of the supports (data not shown), which indicates the irreducibility of the supports. TPR-H_2_ profiles of catalysts are presented in Figure 4.

For the fresh catalyst precursors, two regions of reduction were observed at 125–250 °C and 250–475 °C. Preliminary analysis of these profiles shows that maxima of reduction of active phase precursors deposited on inert silicalite-1 and modified silicalite-1 occurred at different temperatures and the peak intensities depended on the type of supports used. This is a result of differences in the strength of the interaction between precursors and the surface of the above supports that can influence the dispersion of precursor on the supports.

The TPR-H_2_ profiles of Ir/Sil-D catalysts show the main peak in the 125–250 °C range. This peak can be attributed to the reduction of highly dispersed iridium species coming from the reduction of precursor to metallic iridium [19,27,28]. The degree of reduction of the iridium precursor calculated on the basis of hydrogen consumption was 77.6% for the peak with a maximum at 173 °C (Table 4). In the case of catalysts supported on modified silicalite-1 supports the main peak is also present in the 125–250 °C range, but the maximum of peaks is shifted to higher temperatures. For comparison, the TPR-H_2_ studies of iridium precursor impregnated on quartz sand was done showing that such supported hydrogen hexachloroiridate is reduced at 190 °C—inset in Figure 4. The absence of the reduction peaks above 245 °C in the TPR-H_2_ profile of the precursor (H_2_IrCl_6_) indicates the differences in metal-support interactions in comparison to interaction for investigated catalysts as well as the absence of larger clusters of iridium supported on quartz sand (Figure 4, inset). The only peak at 190 °C is observed in the reference sample, which indicates that the iridium precursor is fully reduced at this temperature [29]. The signals in the 245–475 °C region present in the TPR-H_2_ profiles of investigated catalysts are assigned to larger clusters of iridium or to iridium stronger bonded to the support surface [19]. The reduction of the iridium phase supported on both modified silicalite-1 in this region takes place at a lower temperature in comparison to the temperature of reduction for Ir/Sil-D. The maxima of reduction are shifted to lower temperature in the following order Ir/Sil-F-D < Ir/Sil-OH-D < Ir/Sil-D and they are 343, 369, and 398 °C, respectively. It indicates that the temperature used to activate the catalysts (pure H_2_, 400 °C, 2 h) is sufficient for the reduction of the active phase precursor to the metallic phase. The degree of reduction at the higher temperature range depends on the type of supports used and is the lowest for Ir/Sil-D (22.2%) and the highest for Ir/Sil-F-D sample (34.1%) with the highest contribution of medium and strong acid sites. The above-presented data indicates that the contribution of the high-temperature peak of reduction increases with the increasing acidity of the support used (Table 1 and Table 2 and Figure 3). A similar observation was presented by D’Ippolito et al. [25] for Ir/Al_2_O_3_-SiO_2_ systems. The TPR-H_2_ data are in good agreement with the N_2_ adsorption/desorption results (decrease of the volume of low-partial pressure hysteresis loop) indicating the interaction of iridium precursor with the formed defects acting as acid sites.

Another important parameter characterizing metallic catalysts is the dispersion of the active phases. The amount of chemisorbed hydrogen permits the determination of the average size of Ir particles and also the number of iridium surface atoms that is required for the calculation of turnover frequency (TOF, min^−1^) of toluene hydrogenation—Table 5.

The results of hydrogen chemisorption measurements indicate that the textural and acidic properties of supports have an influence on the dispersion and particle size of the iridium active phase. The Ir/Sil-F and Ir/Sil-OH catalysts show much higher dispersion than catalyst supported on unmodified silicalite-1 (Table 5). The highest dispersion was observed for Ir/Sil-OH catalyst (D~34%) with the highest surface area and pores size as well as with high acidity of medium strength. This catalyst exhibits slightly higher dispersion and lower particle size (3.3 nm) when compared to iridium catalyst supported on Sil-F (D~29%, Ir particle size = 3.8 nm). The last one has a higher contribution of stronger acid sites, in comparison to the Sil-OH support, that interacts stronger with iridium precursor causing the formation of bigger Ir particles. Using the modified silicalite-1 supports with mesoporous structure allows to attain higher dispersion and lower particle size compared to iridium catalysts supported on pristine silicalite-1 support, where dispersion was ~22% and Ir particle size ~5 nm. It indicates that the mesoporous structure and large surface area of the modified supports have a crucial effect on the metal dispersion. The acidity of the support has a less significant influence on dispersion and the size of iridium particles.

**Figure 5:**
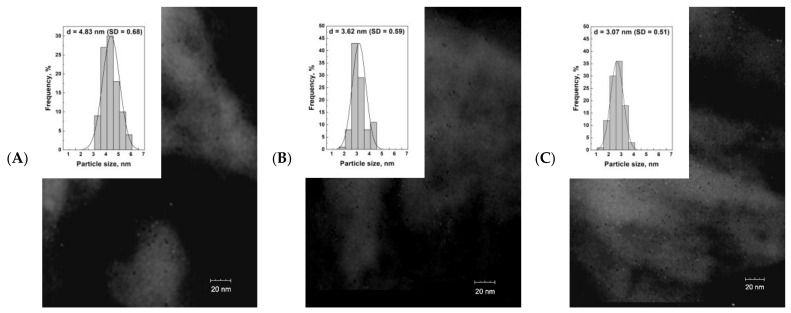
Transmission electron microscope images of Ir/Sil (**A**), Ir/Sil-F (**B**), and Ir/Sil-OH (**C**) catalysts and corresponding particle size distribution (SD) histograms.

The particle size was also estimated by TEM analysis—Figure 5. The TEM images and particle size distribution histograms show that the iridium crystallite size for Ir/Sil (Figure 5A) is almost 50% larger than for the iridium particles supported on the modified silicalite-1 samples. The presented pictures also confirm the homogeneous dispersion of the nanoparticles on the surface of all supports.

The TPD-NH_3_ studies for our supports (Sil-F and Sil-OH—Table 1) indicated a higher number of stronger acid centers for Sil-F material. The lower acidity of the Sil-OH surface favors a slightly better dispersion of the iridium active phase. A similar observation was presented in the literature for modified amorphous silica [17]. The fact that iridium species are weakly bonded to the unmodified support due to its low acidity must also be considered. The presented H_2_ chemisorption results are in agreement with TPR-H_2_ data showing the lowest reduction degree in the 245–475 °C range for Ir/Sil catalyst (Table 5). As a consequence, iridium species show easy migration during the reduction step of the catalyst preparation by forming bigger iridium particles [30].

**Figure 6:**
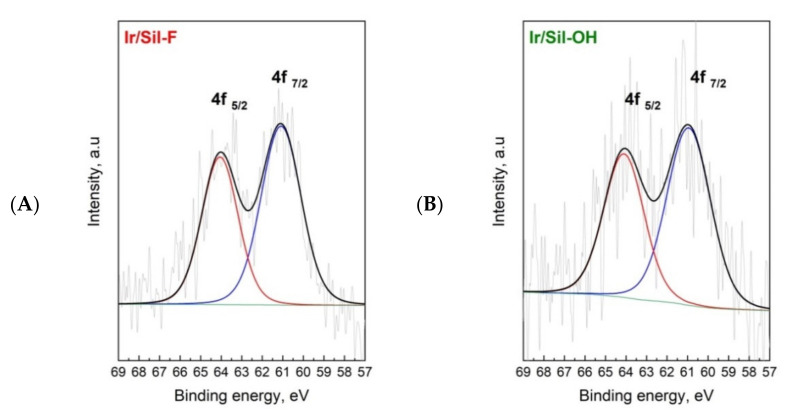
X-ray photoelectron spectra for Ir/Sil-F (**A**) and Ir/Sil-OH (**B**) catalysts after reduction at 400 °C.

The XPS analysis of the Ir 4f spectra of reduced iridium catalysts supported on modified silicalite-1 is shown in Figure 6. The Ir 4f spectra can be fitted into one doublet. The peaks at ~61 and ~64 eV for Ir 4f_7/2_ and Ir 4f_5/2_ were assigned to iridium in the zero valent state [31,32]. There are no peaks at ~62 and ~65 eV attributed to the binding energies of the high valence state of iridium [32,33,34]. It indicates that all iridium phase is reduced to the metallic form.

**Figure 7:**
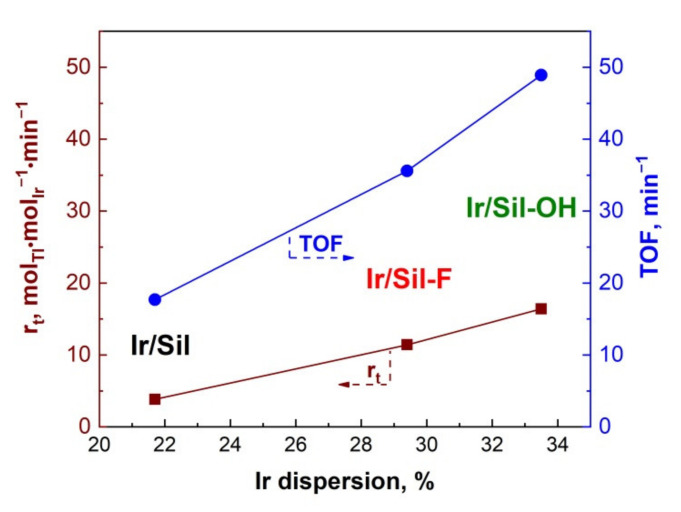
The effect of iridium dispersion on the apparent rate (r_t_) and turnover frequency (TOF) of hydrogenation of toluene.

The catalytic activity of the iridium catalysts has been tested in the hydrogenation of toluene. As we mentioned before, the amount of chemisorbed hydrogen permits to determine the number of iridium surface atoms that is required for the calculation of turnover frequency (TOF, min^−1^) of toluene hydrogenation. Figure 7 shows the dependence of apparent rate (r_t_) and TOF on iridium dispersion. Calculations of r_t_ and TOF have shown that the most active catalyst was Ir/Sil-OH, whose activity was almost 35% higher than that of Ir/Sil-F and was more than 2.5 times higher than the activity of Ir/Sil. The activity of the investigated catalysts, presented as r_t_ as well as TOF, increased with the dispersion of the active phase.

**Figure 8:**
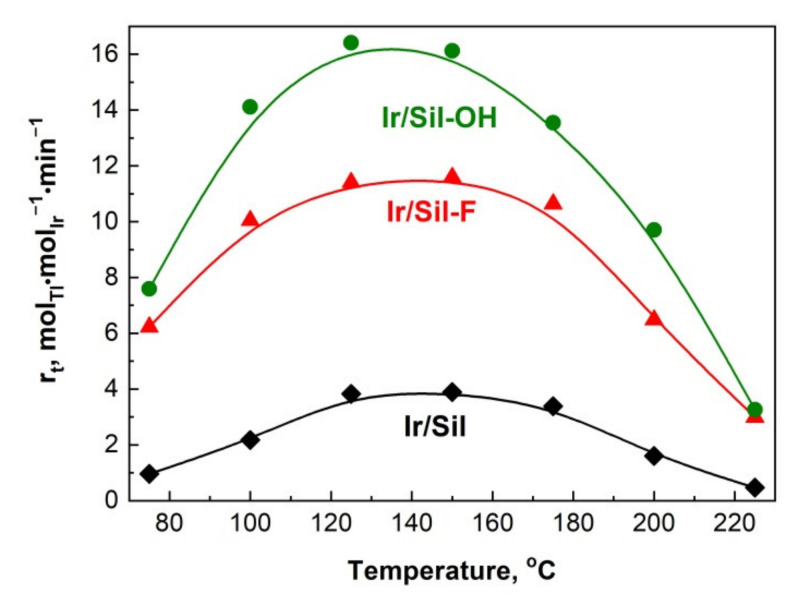
The influence of support on the apparent rate (r_t_) of hydrogenation of toluene as a function of temperature.

The results of the hydrogenation of toluene performed at different temperatures are presented in Figure 8. The catalytic measurements carried out on the supports alone proved no activity in the hydrogenation of toluene.

The activity of the iridium catalyst supported on silicalite-1 modified with NH_4_OH expressed as the apparent rate (r_t_) of reaction (Figure 8) was higher in comparison to the activity obtained over the catalyst supported on Sil-F and unmodified silicalite-1 in all investigated temperatures. The activity increased with the rise of the reaction temperature reaching a maximum at 125 °C for Ir/Sil-OH and 150 °C for Ir/Sil-F as well as Ir/Sil catalysts. For all catalysts, a further increase in temperature caused a decrease in the activity. The decrease of the catalytic activity at higher temperatures is explained in the literature by the occurrence of dehydrogenation [35] or/and cracking of the product of the hydrogenation of toluene—methylcyclohexane [36]. We did not observe any cracking products so the only possible explanation of the decrease in the catalytic activity at 150 °C was the dehydrogenation of methylcyclohexane, which was the only reaction product observed. The Wheeler-Weisz modulus (φ^2^η) [37] estimated for all investigated catalysts were lower than 1 within the temperature range used in this work, allowing to ignore diffusional limitations in applied experimental conditions and their influence on the activity of the investigated catalysts.

Our earlier studies indicated the weak Lewis acid sites on the surface of silicalite-1 modified with NH_4_OH, whereas the modification with NH_4_F caused the generation of strong Lewis acid sites [16]. The presence of weak Lewis acid centers has a positive effect on the hydrogenation of toluene facilitating the adsorption and activation of the aromatic ring. It is in agreement with the findings of Lin et al. [38] showing that the conversion of aromatics on the metal/acid catalysts takes place on two kinds of sites: metal active center and a neighboring acid site on which the adsorbed aromatics can be hydrogenated with the activated hydrogen spillover from metal centers.

**Figure 9:**
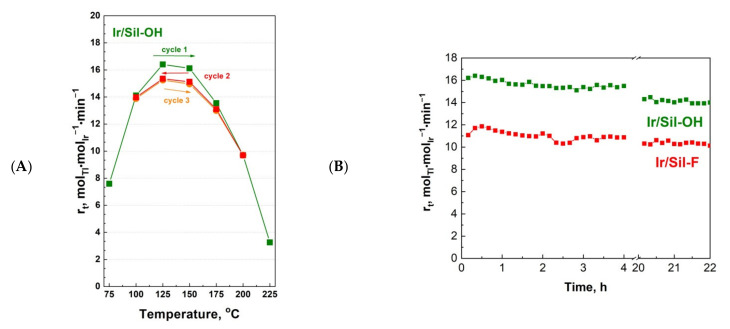
The catalytic activity as a function of the process temperature for the three reaction runs (75 ↗ 225 °C cycle 1; 225 ↘ 100 °C cycle 2; 100 ↗ 200 °C cycle 3) carried out on Ir/Sil-OH catalyst (**A**) and changes in the activity of Ir/Sil-OH and Ir/Sil-F catalysts in toluene hydrogenation as a function of time at 125 °C (**B**).

Figure 9A presents a plot of catalytic activity as a function of the process temperature for the three reaction runs carried out on the most active catalyst, Ir/Sil-OH. The same catalyst was used in three cycles of measurements, with changing the temperature in the range of 75 ↗ 225 °C in cycle 1, 225 ↘ 100 °C in cycle 2, and 100 ↗ 200 °C in cycle 3. At each temperature two measurements were performed at 10 min intervals—the plot was made using the averaged values. As follows from the plot, only in the second cycle a small decrease in the activity was observed in the temperature range of 175–125 °C. This decrease did not exceed 10% of the initial activity, which means that the activity was maintained at a stable level. The catalyst activity in the third cycle was almost the same as that in the second cycle. A similar decrease in activity was observed for Ir/Sil-OH catalyst in the 22 h stability test (Figure 9B). The most active catalysts (Ir/Sil-OH and Ir/Sil-F) were tested at 125 °C for a long period of time (22 h) to check their stability. In the evaluated period of time, the tested catalysts showed stable work. The activity of both catalysts increased for the first 30–40 min of the reaction to the level of ~16.5 and 11.5 mol_Tl_⋅mol_Ir_^−1^⋅min^−1^, respectively, and then it gradually decreased and stabilized after a few hours. The activity of Ir/Sil-OH in the 22 h test decreased by less than 10%, while the activity of Ir/Sil-F fell by 15%.

A comparison of the TOF (48.9 min^−1^) values obtained for the best Ir/Sil-OH catalyst with the results reported in the literature for iridium supported on different supports (SBA-3, Al_2_O_3_, SiO_2_ [39,40], MgF_2_ [19], magnesium oxo-fluoride [41,42]) shows that the iridium catalyst supported on modified silicalite-1 is a more promising catalyst—Table 6.

Based on the paper [43], presenting the influence of different active phases (Pt, Ir, Ru) on catalytic activity for toluene hydrogenation, it can be concluded that iridium may have a great potential for the hydrogenation of aromatic compounds.

## 4. Conclusions

1. The effect of acid properties of modified silicalite-1 supports on the activity of iridium catalysts for toluene hydrogenation was demonstrated.
2. The modification of silicalite-1 caused changes in its structure leading to the production of a better porous structure. New mesopores are formed in the Sil-1 structure and the specific surface area increases significantly, especially after modification with NH_4_OH.
3. Modification of silicalite-1 by treatment with ammonium compound solutions caused the formation of surface acidity (Lewis acid sites) playing a significant role in toluene hydrogenation reaction.
4. Using modified silicalite-1 materials with high surface areas and big pore sizes as supports for Ir catalysts allowed to obtain catalysts with higher Ir dispersion in comparison to iridium catalyst supported on unmodified silicalite-1. It has a crucial effect on the activity for hydrogenation of toluene.
5. Iridium catalyst supported on the silicalite-1 modified by NH_4_OH (Ir/Sil-OH) has excellent catalytic properties for hydrogenation of toluene to methylcyclohexane. The catalyst was also characterized by very stable work.

## Figures and Tables

**Table 1:** Physicochemical characterization of supports and catalysts.

Sample	Activation	S_BET_, m^2^·g^−^^1^	Cumulative Pore Volume, cm^3^·g^−^^1^	Average Pore Diameter ^(a)^, nm	Average Pore Diameter ^(b)^, nm	Total Number of Acid Sites, μmol·g^−1^
Sil	Calcination (air, 3 h, 550 °C)	311	0.18	2.3	2.4	44
Sil-OH		358	0.28	3.2	8.4	188
Sil-F		350	0.24	2.7	5.7	287
Ir/Sil	Reduction (H_2_, 2 h, 400 °C)	319	0.17	2.2	2.3	47
Ir/Sil-OH		359	0.26	2.9	7.2	192
Ir/Sil-F		355	0.23	2.6	6.1	299

^(a)^ pore diameter determined by Brunauer–Emmet–Teller method, ^(b)^ pore diameter determined by Barrett–Joyner–Halenda method from the desorption branch of the isotherm

**Table 2:** The number of acid sites evaluated by temperature-programmed desorption of NH_3_ for iridium catalysts.

Sample	Concentration of Acid Sites, μmol⋅g^−1^			Total Number of Acid Sites, μmol⋅g^−1^
	Weak T ≤ 310 °C	Medium 310–360 °C	Strong T ≥ 360 °C	
Ir/Sil	16	31	-	47
Ir/Sil-OH	97	95	-	192
Ir/Sil-F	56	115	128	299

**Table 3:** X-ray photoelectron spectroscopy results for initial and modified silicalite supports.

	Sil			Sil-OH			Sil-F		
	BE, eV	FWHM	At.%	BE, eV	FWHM	At.%	BE, eV	FWHM	At.%
Si 2p	-	-	-	101.3	2.05	2.6	100.9	1.95	0.9
	103.55	2.1	39.45	103.4	1.96	37.4	103.6	2.13	41.1
Total Si	-	-	39.45	-	-	40.0	-	-	42.1
O 1s	-	-	-	530.6	2.38	2.8	530.8	2.02	3.3
	532.8	2.0	60.12	532.8	2.08	57.2	533.0	2.1	54.6
Total O	-	-	60.12	-	-	60.01	-	-	57.9
Si/O	-	-	0.66	-	-	0.67	-	-	0.73

BE—binding energy, FWHM—full width at half maximum.

**Table 4:** Degree of reduction of iridium catalysts.

Sample	Degree of Reduction, %			2nd Peak /1st Peak Ratio ^(a)^
	Total	125–250 °C	245–475 °C	
		1st Peak	2nd Peak	
Ir/Sil-D	99.8	77.6	22.2	0.29
Ir/Sil-OH-D	97.4	72.1	25.3	0.35
Ir/Sil-F-D	98.6	64.5	34.1	0.53
Ir-precursor/quartz sand	100	100	0	-

^(a)^ Ratio of peak surface at 245–475 °C range to the peak surface at 100–250 °C range.

**Table 5:** Hydrogen chemisorption and toluene hydrogenation activity of iridium catalysts reduced at 400 °C.

Sample	Hydrogen Chemisorption Data for Ir/Support Catalysts ^(a)^					Toluene Hydrogenation ^(c)^ at 125 °C TOF, min^−1^
	Volume Adsorbed, cm^3^·g^−1^			Dispersion, %	Average Size of Ir Particle ^(b)^, nm	
	H_t_	H_irr_	H_r_	D_t_		
Ir/Sil	0.13	0.12	0.01	22	5.1	17.7
Ir/Sil-OH	0.20	0.13	0.06	34	3.3	48.9
Ir/Sil-F	0.17	0.15	0.02	29	3.8	35.6

^(a)^ Dispersion and average size of Ir particles were determined by H_2_ chemisorption; H_t_—total adsorbed hydrogen; H_r_—reversibly adsorbed hydrogen; H_irr_—irreversibly adsorbed hydrogen; D_t_—dispersion calculated from total adsorbed hydrogen. ^(b)^ The average size of iridium particles (in nm) calculated from the amount of total chemisorbed H_2_. ^(c)^ Catalytic activity was expressed as turnover frequency (TOF, min^−1^) in moles of toluene reacted per surface metal atoms (determined by hydrogen chemisorption).

**Table 6:** The activity of iridium catalysts supported on different oxide and non-oxide supports.

Catalysts	Catalysts Activation	Reaction Temperature, °C	TOF, min^−1^	Refs.
Ir/Sil-OH	H_2_ reduction (2 h, 400 °C)	125	48.9	This work
Ir/SBA-3	H_2_ reduction (2 h, 400 °C)	15	38.1	[39]
Ir/Al_2_O_3_	H_2_ reduction (2 h, 400 °C)	150	28.3	[39]
Ir/SiO_2_	H_2_ reduction (2 h, 400 °C)	150	29.4	[39]
Ir/MgF_2_	H_2_ reduction (2 h, 400 °C)	125	15.4	[19]
Ir/MgF_2_-MgO	H_2_ reduction (2 h, 500 °C)	125	12.0	[41]
Ir/Al_2_O_3_	H_2_ reduction (2 h, 377 °C)	125	22.2	[43]
Ir/MgO	H_2_ reduction (2 h, 500 °C)	125	6.0	[41]
Ir/Al_2_O_3_	H_2_ reduction (400 °C)	60	0.6	[40]
Ir/SiO_2_	H_2_ reduction (400 °C)	60	1.1	[40]

## Data Availability

The data presented in this study are available on request from the corresponding author.

## Supplementary Materials

The following are available online at https://www.mdpi.com/article/10.3390/ma14164465/s1. Detailed experimental procedure—TPR-H_2_ and TPD-NH_3_ analysis, H_2_ chemisorption analysis and toluene hydrogenation reaction. Figure S1: A scheme of the setup for catalytic hydrogenation of toluene. Figure S2: XRD patterns of the indicated samples.

## References

[1] Sidhpuria K.B., Parikh P.A., Bahadur P., Tyagi B., Jasra R.V. (2009). Influence of the surface acidity of ZSM-5 support on the catalytic activity of Rh/ZSM-5 for hydrodearomatization of toluene. Catal. Today.

[2] Boricha A.B., Mody H.M., Bajaj H.C., Jasra R.V. (2006). Hydrogenation of benzene over ruthenium-exchanged montmorillonite in the presence of thiophene. Appl. Clay Sci..

[3] Barbier J., Lamy-Pitara E., Marecot P., Boitiaux J.P., Cosyns J., Verna F. (1990). Role of Sulfur in Catalytic Hydrogenation Reactions. Adv. Catal..

[4] Göhlich M., Böttcher S., Räuchle K., Reschetilowski W. (2011). Influence of platinum dispersion on the hydrodearomatization of toluene to light alkanes on Pt/H-ZSM-5. Catal. Commun..

[5] Yasuda H., Sato T., Yoshimura Y. (1999). Influence of the acidity of USY zeolite on the sulfur tolerance of Pd–Pt catalysts for aromatic hydrogenation. Catal. Today.

[6] Tang T., Yin C., Wang L., Ji Y., Xiao F.-S. (2008). Good sulfur tolerance of a mesoporous Beta zeolite-supported palladium catalyst in the deep hydrogenation of aromatics. J. Catal..

[7] Simon L.J., van Ommen J.G., Jentys A., Lercher J.A. (2002). Sulfur tolerance of Pt/mordenites for benzene hydrogenation: Do Brønsted acid sites participate in hydrogenation?. Catal. Today.

[8] Venezia A.M., Parola V.L., Pawelec B., Fierro J.L.G. (2004). Hydrogenation of aromatics over Au-Pd/SiO_2_-Al_2_O_3_ catalysts; support acidity effect. Appl. Catal. A Gen..

[9] Song C., Ma X.L. (2003). New design approaches to ultra-clean diesel fuels by deep desulfurization and deep dearomatization. Appl. Catal. B Environ..

[10] Castaño P., Pawelec B., Aguayo A.T., Gayubo A.G., Arandes J.M. (2008). The Role of Zeolite Acidity in Coupled Toluene Hydrogenation and Ring Opening in One and Two Steps. Ind. Eng. Chem. Res..

[11] Lee J.K., Rhee H.K. (1998). Sulfur tolerance of zeolite beta-supported Pd−Pt catalysts for the isomerization of n-hexane. J. Catal..

[12] Chupin J., Gnep N.S., Lacombe S., Guisnet M. (2001). Influence of the metal and of the support on the activity and stability of bifunctional catalysts for toluene hydrogenation. Appl. Catal. A.

[13] Grzechowiak J.R., Szyszka I., Rynkowski J., Rajski D. (2003). Preparation, characterisation and activity of nickel supported on silica-titania. Appl. Catal. A.

[14] Janiszewska E., Macario A., Wilk J., Aloise A., Kowalak S., Nagy J.B., Giordano G. (2013). The role of the defect groups on the Silicalite-1 zeolite catalytic behawior. Micropor. Mesopor. Mat..

[15] Bonelli B., Forni L., Aloise A., Nagy J.B., Fornasari G., Garrone E., Gedeon A., Giordano G., Trifirò F. (2007). Beckmann rearrangement reaction: About the role of defect groups in high silica zeolite catalysts. Micropor. Mesopor. Mater..

[16] Janiszewska E., Kowalska-Kuś J., Góra-Marek K., Szymocha A., Nowińska K., Kowalak S. (2019). Modification of silicalite-1 with ammonium compounds aimed at preparation of acidic catalyst for acetalization of glycerol with acetone. Appl. Catal. A.

[17] Janiszewska E., Kot M., Zieliński M. (2018). Modification of silica with NH^4+^ agents to prepare an acidic support for iridium hydrogenation catalyst. Micropor. Mesopor. Mat..

[18] Moreau C., Geneste P., Moffat J.B. (1990). Theoretical Aspects of Heterogeneous Catalysis.

[19] Zieliński M., Pietrowski M., Wojciechowska M. (2011). New Promising Iridium Catalyst for Toluene Hydrogenation. ChemCatChem.

[20] Siang J.Y., Lee C.C., Wang C.H., Wang W.T., Deng C.Y., Yeh C.T., Wang C.B. (2010). Hydrogen production from steam reforming of ethanol using a ceria-supported iridium catalyst: Effect of different ceria supports. Int. J. Hydrogen Energy.

[21] Malhis A.A., Arar S.H., Fayyad M.K., Hodali H.A. (2018). Amino- and thiol-modified microporous silicalite-1 and mesoporous MCM-48 materials as potential effective adsorbents for Pb(II) in polluted aquatic systems. Adsorpt. Sci. Technol..

[22] Thommes M., Kaneko K., Neimark A.V., Olivier J.P., Rodriguez-Reinoso F., Rouquerol J., Sing K.S.W. (2015). Physisorption of gases, with special reference to the evaluation of surface area and pore size distribution (IUPAC Technical Report). Pure Appl. Chem..

[23] Tao Y., Kanoh H., Kaneko K. (2005). Comment: Questions Concerning the Nitrogen Adsorption Data Analysis for Formation of Supermicropores in ZSM-5 Zeolites. Adv. Mater..

[24] Li Y., Zhang W., Zhang L., Yang Q., Wie Z., Feng Z., Li C. (2004). Direct Synthesis of Al–SBA-15 Mesoporous Materials via Hydrolysis-Controlled Approach. J. Phys. Chem. B.

[25] D’Ippolito S.A., Ballarini A.D., Pieck C.L. (2017). Influence of Support Acidity and Ir Content on the Selective Ring Opening of Decalin over Ir/SiO_2_–Al_2_O_3_. Energy Fuel..

[26] Alfonsetti R., Lozzi L., Passacantando M., Picozzi P., Santucci S. (1993). XPS studies on SiOx thin films. Appl. Surf. Sci..

[27] Moreno-Tost R., Rodríguez-Castellón E., Jiménez-López A. (2006). Cobalt–iridium impregnated zirconium-doped mesoporous silica as catalysts for the selective catalytic reduction of NO with ammonia. J. Mol. Catal. A Chem..

[28] Li L., Zhang F., Guan N., Richter M., Fricke R. (2007). Selective catalytic reduction of NO by propane in excess oxygen over IrCu-ZSM-5 catalyst. Catal. Commun..

[29] Subramanian S., Schwarz J.A. (1991). Stoichiometric composition of platinum, iridium and platinum-iridium catalytic precursors. Appl. Catal..

[30] Mironenko R.M., Belskaya O.B., Talsi V.P., Gulyaeva T.I., Kazakov M.O., Nizovskii A., Kalinkin A.V., Bukhtiyarov V.I., Lavrenov A.V., Likholobov V.A. (2014). Effect of γ-Al_2_O_3_ hydrothermal treatment on the formation and properties of platinum sites in Pt/γ-Al_2_O_3_ catalysts. Appl. Catal. A Gen..

[31] Hara M., Asami K., Hashimoto K., Masumoto T. (1983). An X-ray photoelectron spectroscopic study of electrocatalytic activity of platinum group metals for chlorine evolution. Electrochim. Acta.

[32] Nakagawa K., Ikenaga N., Suzuki T., Kobayashi T., Haruta M. (1998). Partial oxidation of methane to synthesis gas over supported iridium catalysts. Appl. Catal. A Gen..

[33] Fonscea G.S., Machado G., Teixeira S.R., Fecher G.H., Morais J., Alves M.C.M., Dupont J. (2006). Synthesis and characterization of catalytic iridium nanoparticles in imidazolium ionic liquids. J. Colloid Interf. Sci..

[34] Nassreddine S., Massin L., Aouine M., Geantet C., Piccolo L. (2011). Thiotolerant Ir/SiO_2_–Al_2_O_3_ bifunctional catalysts: Effect of metal–acid site balance on tetralin hydroconversion. J. Catal..

[35] Peyrovi M.H., Toosi M.R. (2008). Study of benzene hydrogenation catalyzed by nickel supported on alumina in a fixed bed reactor. React. Kinet. Catal. Lett..

[36] Loiha S., Föttinger K., Zorn K., Klysubun W., Rupprechter G., Wittayakun J. (2009). Catalytic enhancement of platinum supported on zeolite beta for toluene hydrogenation by addition of palladium. J. Ind. Eng. Chem..

[37] Weisz P.B., Prater D.C. (1954). Interpretation of measurements in experimental catalysis. Adv. Catal..

[38] Lin S.D., Vannice M.A. (1993). Hydrogenation of Aromatic Hydrocarbons over Supported Pt Catalysts. I. Benzene Hydrogenation. J. Catal..

[39] Kiderys A., Kot M., Janiszewska E., Pietrowski M., Yang C.-M., Zieliński M. (2020). SBA materials as support of iridium catalyst for hydrogenation reactions. Catal. Today.

[40] Cunha D.S., Cruz G.M. (2002). Hydrogenation of benzene and toluene over Ir particles supported on γ-Al_2_O_3_. Appl. Catal. A.

[41] Zieliński M., Kiderys A., Pietrowski M., Tomska-Foralewska I., Wojciechowska M. (2016). Synthesis and characterization of new Mg–O–F system and its application as catalytic support. Catal. Commun..

[42] Zieliński M., Wojciechowska M. (2012). Iridium Supported on MgF_2_-MgO as Catalyst for Toluene Hydrogenation. Catal. Commun..

[43] Zieliński M., Pietrowski M., Kiderys A., Kot M., Alwin E. (2017). A comparative study of the performance of Pt/MgF_2_, Ir/MgF_2_ and Ru/MgF_2_ catalysts in hydrogenation reactions. J. Fluorine Chem..

