# Supplementary material for: Studies of New Iridium Catalysts Supported on Modified Silicalite-1—Their Structure and Hydrogenating Properties
Source: Materials (Basel). 2021 Aug 9;14(16):4465. doi: 10.3390/ma14164465 (PMC8398754; doi:10.3390/ma14164465)
Supplement: Supplementary file 1 [file materials-14-04465-s001.zip › materials-1280845-supplementary.pdf]

materials

Supplementary Materials

Studies of New Iridium Catalysts Supported on Modified Silicalite-1—Their Structure and Hydrogenating Properties

Michał Zieliński [1,*], Monika Kot [1], Mariusz Pietrowski [1], Robert Wojcieszak [2], Jolanta Kowalska-Kuś [1] and Ewa Janiszewska [1,*]

[1] Faculty of Chemistry, Adam Mickiewicz University in Poznań, Uniwersytetu Poznańskiego 8, 61-614 Poznań, Poland; monika.kot@amu.edu.pl (M.K.); mariop@amu.edu.pl (M.P.); jolakow@amu.edu.pl (J.K.-K.)
[2] Univ. Lille, CNRS, Centrale Lille, Univ. Artois, UMR 8181-UCCS-Unité de Catalyse et Chimie du Solide, F-59000 Lille, France; robert.wojcieszak@univ-lille.fr
* Correspondence: mardok@amu.edu.pl (M.Z.); eszym@amu.edu.pl (E.J.)

Detailed Experimental Procedure:

Temperature-programmed reduction with hydrogen (TPR-H_2) analysis

Dried metal precursor-impregnated supports (~50 mg) were reduced in the flow of 10 vol.%. H_2–Ar (99.999%, Linde, Pullach im Isartal, Germany) at the flow rate of 30 $cm^3 \cdot min^{-1}$. The measurements were conducted in the temperature range from 50 to 700 °C at a linear temperature ramp of 10 °C·min^{-1}. In the TPR-H_2 studies, a quartz sand (Aldrich, Saint Louis, MO, USA) impregnated with metal precursors ($H_2IrCl_6 \times H_2O$) was used as a reference material. The products of the reduction were retained by an isopropanol/liquid nitrogen cold trap at about −70 °C. The signal intensity was normalized to 100 mg for each sample studied.

Temperature-programmed desorption of ammonia (TPD-NH_3) analysis

In the typical experiment a sample (~50 mg) was heated in He (99.999%, Linde, Pullach im Isartal, Germany) at the rate of 10 °C min^{-1} up to 400 °C and then maintained at this temperature for 60 min. Afterwards, it was cooled down to 120 °C and saturated with ammonia for 30 min. The physically adsorbed NH_3 was removed by purging with helium flow at 120 °C for 1 h. Measurements of temperature-programmed desorption were conducted in helium flow (30 $cm^3 \cdot min^{-1}$) at a heating rate of 10 °C·min^{-1} to 400 °C and hold for 30 min at 400 °C. The desorbing ammonia was monitored with a thermal conductivity detector (TCD) and the signal intensity was normalized to 1 g for each sample studied. The calibration was performed by dispensing 1 cm^3 NH_3 from a calibrated gas loop into the helium flow.

H_2 chemisorption analysis

The samples were evacuated for 10 min at room temperature, then at 360 °C for 30 min, followed by reduction in hydrogen flow (40 $cm^3 \cdot min^{-1}$) at 360 °C for 30 min and evacuation at 360 °C for 60 min. The additional treatment in the flow of hydrogen was aimed to clean the sample from impurities that could adsorb on the sample surface during its contact with atmospheric air. Chemisorption of hydrogen was carried out at 35 °C and the isotherms were determined using 5 different pressures in the range of 12–40 kPa. By assuming the stoichiometry of one hydrogen atom per one surface iridium atom (Ir_s), the dispersion of iridium (D) can be expressed as $D = Ir_s/Ir_t = H/Ir_t$ (where Ir_t–total number of iridium atoms).

Toluene hydrogenation reaction

Fresh dried catalyst (25 mg) was loaded into the reactor and reduced in situ in a flow (100 $cm^3 \cdot min^{-1}$) of pure hydrogen at 400 °C for 2 h before starting the reaction. After reduction the temperature was lowered to 50 °C. The reaction mixture obtained by passing hydrogen (50 $cm^3 \cdot min^{-1}$) through a saturator filled with toluene (Aldrich, Saint Louis, MO, USA) equilibrated at 10 °C was directed to the reactor. The concentration of toluene in the feed was stable and equal to 0.75 $\mu mol \cdot cm^{-3}$. The catalysts were heated at the rate of 10 $°C \cdot min^{-1}$ in a flow of pure hydrogen and the catalytic activities were measured at temperatures between 75 and 225 °C in several steps over the same catalyst. The reaction was carried out for 20 min at each temperature and the products were analyzed every 10 min using a manually sampling system. Relative Molar Factors for toluene and methylcyclohexane were 116 and 120, respectively [1]. These coefficients were used to recalculate peak areas into molar quantities. Toluene was dosed using six-way valve that enabled the mixture to bypass the reactor. This procedure was applied in order to estimate concentration of toluene.

The reaction products were analysed on SRI gas chromatograph (SRI Instruments, Earl St. Torrance, CA, USA) equipped with a capillary column RESTEK MXT-1. The identification of toluene and methylcyclohexane was carried out using a thermal conductivity detector (TCD) detector. A scheme of the setup used for catalytic hydrogenation of toluene is presented in Figure S1.

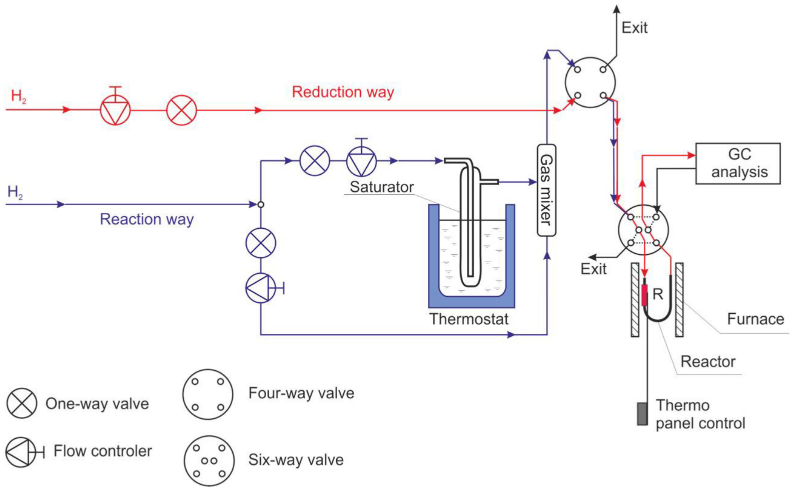

Figure S1. A scheme of the setup for catalytic hydrogenation of toluene. GC—gas chromatography.

Powder X-ray diffraction patterns were recorded in order to confirm the correct MFI structure of supports and catalysts and are presented in Figure S2.

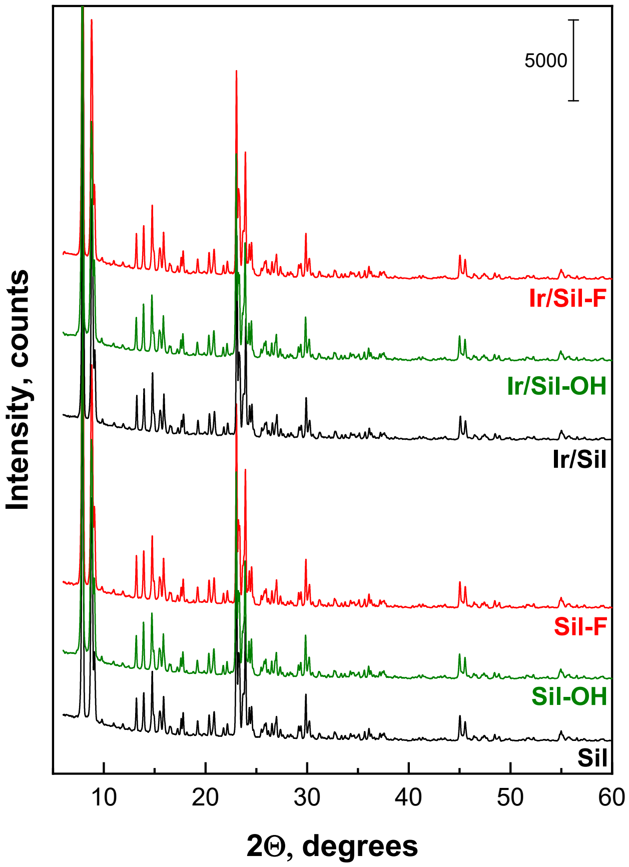

Figure S2. XRD patterns of the indicated samples.

References

1. Dietz, W.A. Response Factors for Gas Chromatographic Analyses. *J. Chromatogr. Sci.* **1967**, *5*, 68–71. doi:10.1093/chromsci/5.2.68.
